# Homology modeling and molecular dynamics dimulation study of β carbonic anhydrase of Ascaris lumbricoides

**DOI:** 10.6026/97320630015572

**Published:** 2019-09-09

**Authors:** Mahima Yadav, Shikha Khandelwal

**Affiliations:** 1 Amity Institute of Biotechnology, Amity University Haryana, Gurgaon-122413, India

**Keywords:** Ascariasis, carbonic anhydrase, structure analysis, homology modeling, Schrodinger software, molecular dynamics

## Abstract

Ascaris lumbricoides is the prevalent parasite causing ascariasis by infecting the human alimentary tract. This is common in the jejunum of small intestine. Therefore, it is of interest to describe the target protein β Carbonic Anhydrase involved in Ascariasis. Carbonic anhydrase (CAs, the metallo enzymes) is encoded by six evolutionary divergent gene families α, β,γ, δ, ζ, and η, which contain zinc ion in their catalytic active site. β-CA is found in plants, algae, fungi, bacteria, protozoans, arthropods, and nematodes and completely absent in vertebrate genomes. The absence of β-CA protein in vertebrate makes the enzyme an important target for inhibitory studies against helminthic infection. The sequence to function related information and 3D structure data for β-CA of Ascaris lumbricoides is not available. Hence, we modeled the 3D structure (using PRIME) for the molecular dynamics and simulation studies (using the Desmond of Schrodinger software) and interaction analysis (using STRING database). The β-CA protein found to be interacting with carbonic anhydrase protein family along with T27A3, alh13, mtp18, T22F3, gcy29 proteins. These results provide insights for the understanding of the functional and biological roles played by β CA. Hence, this data is useful for the design of drugs for Ascariasis.

## Background

Carbonic anhydrases (CAs) have been recently identified as potential targets for novel anti-infective drugs. CA's are encoded by six evolutionary divergent gene families α, β, γ, δ, ζ, and η CAs. All members of these gene families are metallo enzymes containing zinc ion in their catalytic active site [1,2]. α-CAs are the most studied family including 13 catalytically active members in mammals: cytosolic enzymes (CA I, CA II, CA III, CA VII, and CA XIII), membrane-bound (CA IV, CA IX, CA XII, CA XIV, and CA XV), mitochondrial CAs (VA and CA VB), and secreted CA (VI). Certain ζ- and γ-CAs contain cadmium (II), iron (II) or cobalt (II) as alternative metal ion cofactors. β-CAs are only found in plants, algae, fungi, bacteria, protozoans, arthropods, and nematodes [3]. The β-CA genes are completely absent in vertebrate genomes and hence they are considered as potential targets for novel antihelminthic diseases [4].

Ascariasis is a disease caused by the parasitic roundworm Ascaris lumbricoides. In more than 85% of cases, infections have no symptoms, especially if they are small in numbers. Ascariasis is endemic disease, mainly found in tropical and temperate regions of the world, where there is sufficient moisture and in areas which have poverty, ignorance and low standard of hygiene and sanitation [5].The clinically approved sulfonamide acetazolamide is the only inhibitor tested on Ascaris lumbricoides β-CA protein, with an inhibition constant of 84.1 nM [2]. But its inability to penetrate the nematode was found to be a major problem. Therefore, various studies have been carried out to improve the penetration efficacy of β-CA inhibitors through biological membranes and cuticles of worms. Therefore to better understand the mechanism of interaction of this protein with drug, there is a need to understand the three dimensional structure and interaction part of this protein. In this study, we modeled the 3D structure of A. lumbricoides β-CA (AlBCA) and also analyzed the different physio chemical and functional properties which would help the researcher to do more advance studies related to function and role of β carbonic anhydrase as a target protein for Ascaris lumbricoides.

## Methodology

### Retrieval of β-CA protein sequence

Protein sequence of the beta carbonic anhydrase of A. lumbricoides was retrieved in FASTA format from the UniProt KB/TrEMBL, with accession no: A0A0M3IN45 and 259 amino acid length [6].

### Prediction of physico-chemical properties

The various physical and chemical parameters such as amino acids composition, molecular weight, theoretical isoelectric point, total number of negative and positive charged residues, extinction coefficient, instability index, aliphatic index and GRAVY were calculated using Expasy's ProtParam server [7].

### Functional characterization and prediction of secondary structure

The motifs or conserved pattern can be identified with the help of Prosite database, Interproscan and SMART database [8]. Secondary structure was predicted using SOPMA tool in Expasy defining the helix, turns and sheet of protein sequence [9].

### 3D Homology modelling and validation

The structure of β-CA protein of A. lumbricoides is not available in PDB. Therefore, the sequence of β-CA was downloaded from UniprotKB (ID: A0A0M3IN45). The suitable template for homology modeling was identified on PDB using the BLAST P program [10]. With the help of identified template protein, the 3D model of β-CAwas built using Prime in Schrodinger Suite 2019-2 [11,12]. The target (β-CA) and template sequences were aligned using the Clustal W method in Prime, followed by manual adjustment to avoid big gaps.The overall stability of the protein was assessed using Ramachandran plot [13].

### Molecular dynamic (MD) simulations

The modelled 3D structure of β-CA was further optimized using MD simulations. The MD simulations were performed using Desmond Molecular Dynamics module [11,12] of Schrodinger. Energy of the prepared systems was minimized for 2000 iterations using steepest descent method. The default parameters in Desmond were applied for system equilibration. The 100 ns (nano seconds) MD simulations were then carried out with the equilibrated systems at a temperature of 300 K and at a constant pressure of 1atm, under the NPT (normal pressure and normal temperature) ensemble with a time step of 2fs (femto seconds).

### Prediction of binding site

Modeled protein structure was used to identify the active binding sites using sitemap program of Schrodinger software.

### Functional analysis and protein-protein interaction study:

STRING v10.0 server [14] was used to find out the β-CA proteinprotein interactions of A. lumbricoides with other closely related proteins and functional protein association network was generated.

## Result and Discussion:

### The protein sequence of β-CA, retrieved from UNIPROT (ID:

A0A0M3IN45) is 259 amino acids in length. The physiochemical properties of β-CA were calculated using ProtParam (Table 1). The amino acid composition indicated the maximum percentage of Leu residues i.e 9.3% and followed by Ala and Lys with 8.5 % and 8.9% respectively. Highest percentage of leucine residue in β-CA might be correlated with presence of leucine switch motif. PI value of 7.33 defines the slightly alkaline behaviour of the protein and instability index 44.09 which is higher than 40 defined its unstable nature. This instability was expected due to presence of certain dipeptides which is different in unstable proteins as compared to stable ones. Higher aliphatic index (81.74) suggested that protein is thermostable and the lower value of GRAVY (-0.328) indicated the possibility of better interaction with water. Extinction coefficient at 280nm is 15470 M-1cm-1 with respect to concentrations of Cys, Trp and Tyr. This coefficient helps in the quantitative study of proteinprotein and protein-ligand interactions in solution.

**Figure 1:**
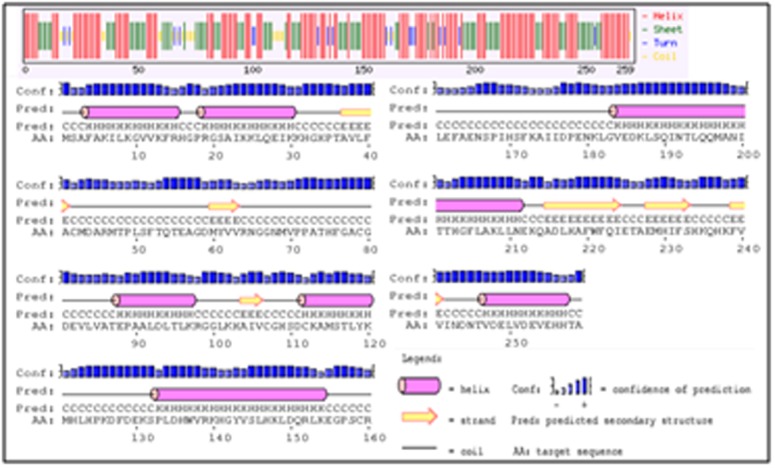
Secondary structure analysis of β-CA protein using SOPMA.

The motifs or conserved pattern was identified in the sequence. The domain Pro_CA was found in the sequence from 30 to 233 in length out of 259 amino acid sequence with E value 8.98e-22.Interproscan result showed protein family membership or homologous superfamily is carbonic anhydrase (IPR001765) and detailed signature matches with PF00484(Pro CA) and SM00947 (Pro CA 2) of carbonic anhydrase. Secondary structure prediction was done by using SOPMA. The result showed that the percentage of alpha helix was more than the sheet and turn (Figure 1). The percentage of alpha helix was found to be 40.15%, beta sheets to be 6.44% and beta turn was to be 8.9%. No disordered protein binding sites were identified in this protein and this 40.15% α-helix makes this protein stable for homology modelling.

**Figure 2:**
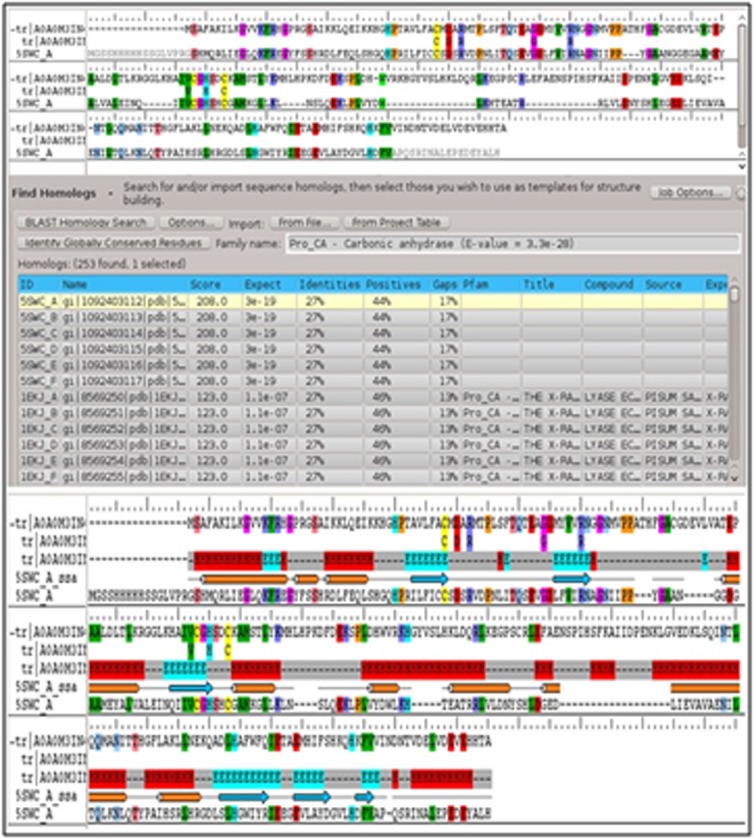
(a) Alignment between target (β-CA) and template (Synechocystis sp. (5SWC A)); (b) Alignment showing H for helix; E for strand; and - for loop.

**Figure 3:**
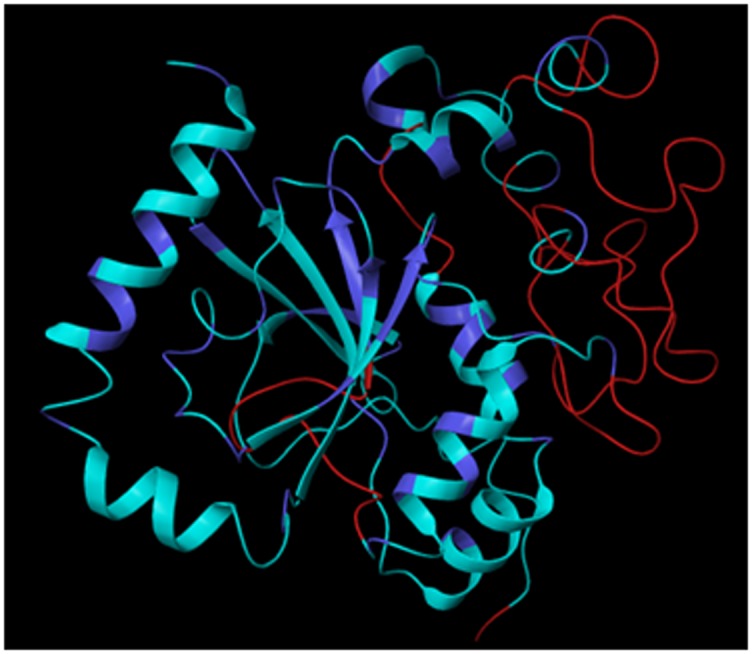
Model of β-CA protein built on the 5SWC _A template.

**Figure 4:**
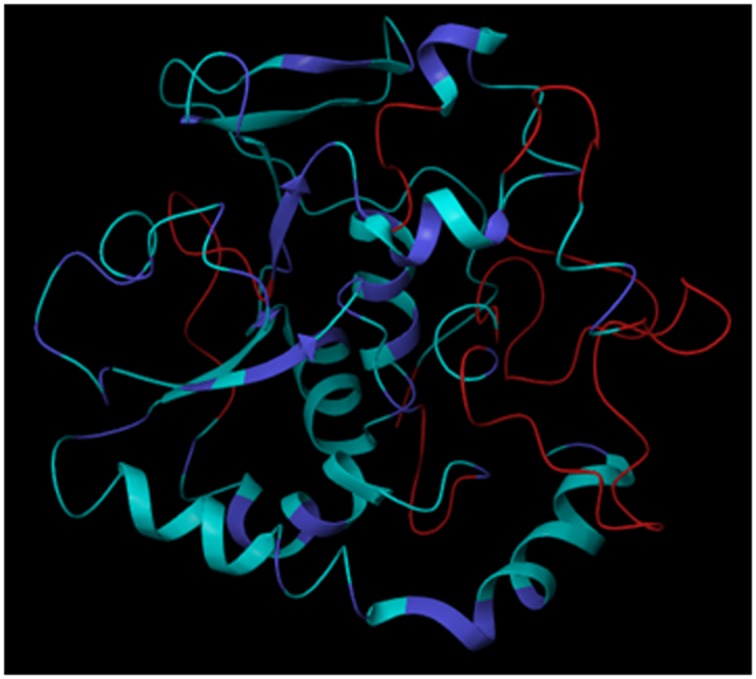
Optimized 3D Model of β-CA protein after 100 ns MD simulations

**Figure 5:**
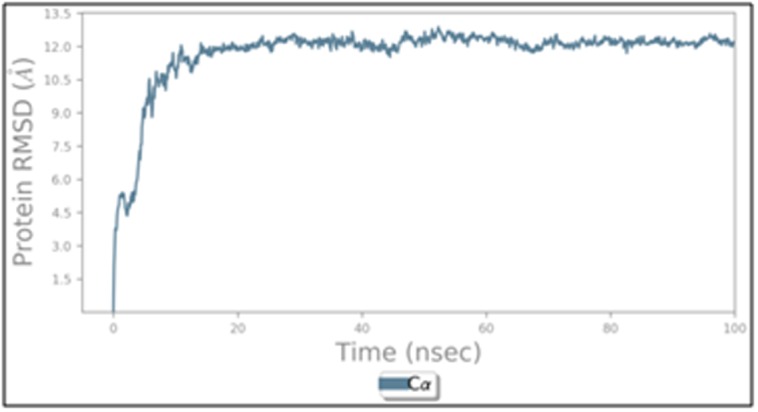
RMSD graph of theβ-CA protein

Since, there were no resolved 3D structures available for the β-CA protein of A. lumbricoides in PDB. In literature, it was reported that for homology modelling, sequence identity between target and template proteins should be 25% and more [15]. So, in this study, an attempt was made to model the 3D structure through homology modelling using BLAST P against known protein structures deposited in PDB. The crystal structure of Synechocystis sp. (5SWC A) was selected with a 27.98% sequence similarity for the target sequence. The sequence alignment between the template (5SWCA) and the target was shown in [Figure 2 (a) and (b)]. Homology modelling was done using the PRIME of Schrodinger Suite 2019-2. The homology model so obtained was further refined by Protein Prep (Protein preparation) wizard in Schrodinger software. This refinement mainly involves stabilization of the protein by hydrogen bond addition, structure optimization and energy minimization. [Figure 3]showed the modelled structure of β-CA and the ribbon diagram of super imposed structures of the template and target, respectively. The build 3D structure of β-CAobtained by homology modelling was further refined and optimized using MD simulation. In MD simulations, RMSD (Root Mean Square Deviation) serves as a measure to determine the stability of β-CA structure based on its deviation from the initial structure. The optimized 3D model was shown in [Figure 4]. The RMSD values of β-CA residues in the entire MD simulation trajectory were shown in [Figure 5].

**Figure 6:**
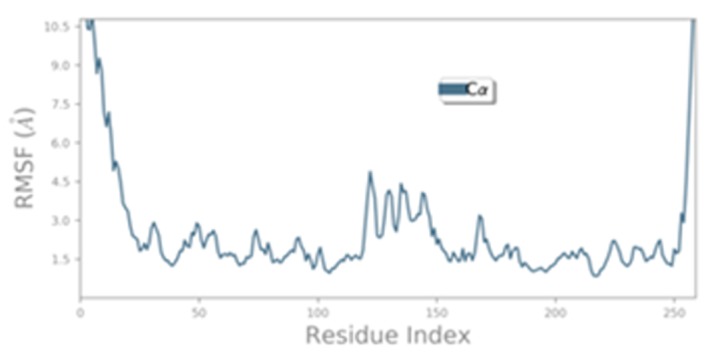
RMSF graph of the β-CA protein

**Figure 7:**
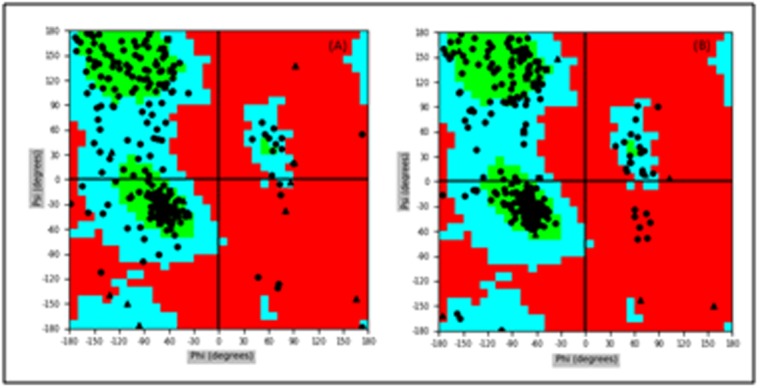
Ramachandran plot of Modelled β-CA protein at 0 ns (A) and 100ns (B), respectively

**Figure 8:**
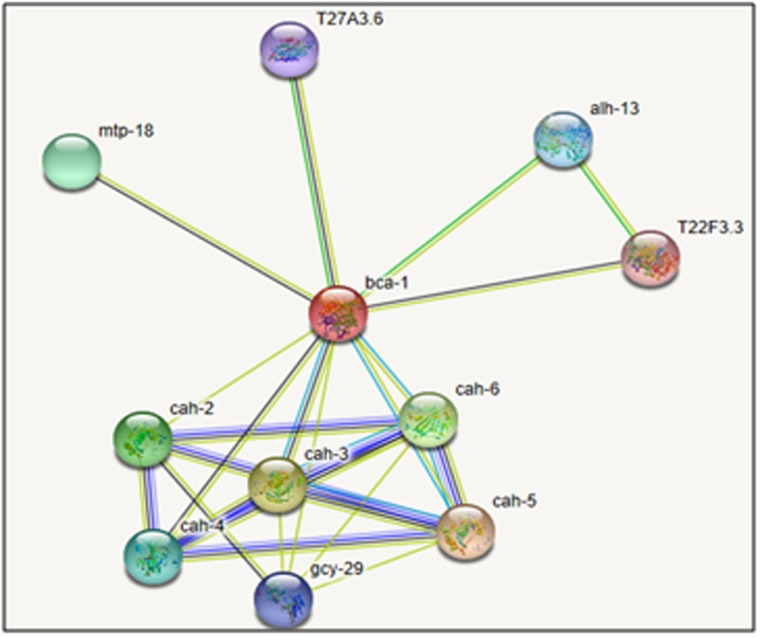
Protein-protein interaction map for the β-CA of A. lumbricoides.

The β-CA structure showed deviations up to 10.5 Å and then gave a stable trajectory beyond that. The 3D structure of β-CA reached to a stable state after 9 sec.[Figure 6] shows RMSF (root mean-square fluctuations) of β-CA structure generated during the MD simulation and these fluctuations were calculated to characterize the mobility of individual residues. On this plot, peaks indicate areas of the protein that fluctuate the most during the simulation. Typically the tails (N-and Cterminal) fluctuate more than any other part of the protein. Secondary structure elements like alpha helices and beta strands are usually more rigid than the unstructured part of the protein, and thus fluctuate less than the loop regions. The fluctuations reported were within 4.5 Å indicating that the amino acid fluctuations were in acceptable range. The stereo chemical quality of the 3D model was validated by Ramachandran plot.[Figure 7] showed that around 97.29% residues were present in the allowed regions (92.66% in the favoured region and 4.6% residues in the allowed regions) and only 2.7% residues were present in the outlier region indicating that the quality of the model was good. Binding sites prediction was also done using sitemap program Table 2. The result showed five binding sites and best binding site was identified with a Dscore of 1.068. Functional analysis revealed ten potential interacting partners of β-CA protein in the protein interaction network as resolved by STRING analysis. The β-CA protein has interaction with Carbonic anhydrase protein family along with T27A3, alh13, mtp18, T22F3, gcy29 protein (Figure 8).

## Conclusion

The 3D structure of the β-CA protein was modelled using PRIME, further optimized by 100 ns molecular dynamic simulations and validated by Ramachandran plot. Detailed data for the structure and function of β-CA is not available till date. Moreover, the absence of β-CAs in vertebrates makes this enzyme as a potential target for various helminthic diseases. The modelled 3D structure of β-CAs is of use for anti-parasitic drug designing using known potential binding sites of protein. This in silico study will help in designing new broad-spectrum and preferably single dose β-CA inhibitors, against Ascaris lumbricoides β-CA.

## Figures and Tables

**Table 1:** Physiochemical properties of β-CA protein using Expasy's ProtParam tool

Length	Molecular weight	Theoretical pI	Aliphatic index	Grand average of hydropathicity (GRAVY)	Instability index	Extinction coefficients	negatively charged residues (Asp +Glu)	positively charged residues (Arg + Lys):
259	29029.4	7.33	81.74	-0.328	44.09	15470	31	31

**Table 2:** Binding sites of β-CA protein

S. No	Title	Site score	Size	D. score	Volume
1	Sitemap_1_site_1	1.068	187	1.054	340.5
2	Sitemap_1_site_2	1.016	177	1.004	337.8
3	Sitemap_1_site_3	0.94	176	0.967	441
4	Sitemap_1_site_4	0.818	76	0.82	205.1
5	Sitemap_1_site_5	0.759	49	0.747	83

## References

[1]  Emameh L (2010). BMC Biochem..

[2]  Zolfaghari Emameh R (2015). Parasit Vectors..

[3]  Rowlett RS (2010). Biochem Biophys Acta..

[4]  Capasso C, Supuran CT (2015). J Enzyme Inhib Med Chem..

[5]  Shah J, Shahidullah A (2018). Case Rep Gastroenterol..

[6]  Bairoch A, Apweiler R (2000). Nucleic Acids Res..

[7]  Gasteiger E (2005). In: John M. Walker (ed.) The Proteomics ProtocolsHandbook, Humana Press..

[8]  Falquet L (2002). Nucl Acids Res..

[9]  Geourjon C, Deleage G (1995). Comput Appl Biosci..

[10]  Marsden RL (2008). Orengo CA. Methods Mol. Biol..

[11]  Jacobson MP (2002). J. Mol. Biol..

[12]  Jacobson MP (2004). Proteins: Structure, Function and Bioinformatics.

[13]  Ramachandran GN (1963). J Mol Biol..

[14]  Szklarczyk (2015). Nucleic Acids Res..

[15]  Yang AS, Honig B (2000). J. Mol. Biol..

