# Correction: Homology modeling and molecular dynamics simulation study of beta carbonic anhydrase of Ascaris lumbricoides in Yadav and Khandelwal, 2019

**DOI:** 10.6026/97320630017528

**Published:** 2021-04-30

**Authors:** Mahima Yadav, Shikha Khandelwal

**Affiliations:** 1 Amity Institute of Biotechnology, Amity University Haryana, Gurgaon-122413, India

Title should read as “Homology modeling and molecular dynamics simulation study of beta carbonic anhydrase of Ascaris lumbricoides” in Yadav and Khandelwal, (2019) Bioinformation. 2019 15:572. PMID: 31719767.

